# Pretreatment nutritional risk scores and performance status are prognostic factors in esophageal cancer patients treated with definitive chemoradiotherapy

**DOI:** 10.18632/oncotarget.21940

**Published:** 2017-10-19

**Authors:** Tao Song, Qiuyan Wan, Wenke Yu, Jianbo Li, Shaohua Lu, Chen Xie, Hongqing Wang, Min Fang

**Affiliations:** ^1^ Department of Radiation Oncology, Zhejiang Provincial People's Hospital, People's Hospital of Hangzhou Medical College, Hangzhou 310000, Zhejiang, P.R. China; ^2^ Department of Gynecologic Oncology, Jiangxi Cancer Hospital, Nanchang 330029, Jiangxi, P.R. China; ^3^ Department of Radiology, Zhejiang Qingchun Hospital, Medical College of Zhejiang University, Hangzhou 310000, Zhejiang, P.R. China; ^4^ Department of Radiation Oncology, Ningbo Mingzhou Hospital, Ningbo 315000, Zhejiang, P.R. China; ^5^ Department of Radiation Oncology, Jinhua Guangfu Hospital, Jinhua 321000, Zhejiang, P.R. China; ^6^ Department of Radiation Oncology, Jiangxi Cancer Hospital, Nanchang 330029, Jiangxi, P.R. China; ^7^ Department of Radiology, The First Affiliated Hospital of Wenzhou Medical University, Wenzhou 325000, Zhejiang, P.R. China

**Keywords:** esophageal cancer, NRS-2002, ECOG PS, treatment response, survival

## Abstract

This study evaluated the prognostic effects of nutritional risk scores and performance status (PS) on unresectable locally advanced esophageal cancer (LAEC) patients who were treated with definitive concurrent chemoradiotherapy (dCRT). A total of 202 LAEC patients from four different cancer centers were retrospectively reviewed. Nutritional risk and PS were measured using the Nutritional Risk Screening 2002 (NRS-2002) scores and Eastern Cooperative Oncology Group (ECOG) scales. Outcomes were clinical response rate, overall survival (OS) and progression-free survival (PFS). Multivariate analysis of predictive factors of response to dCRT and survival were performed using a logistic regression and a Cox model, respectively. The majority of patients (71.8%) had an ECOG PS score of 0-1, and 52.5% (n=106) of patients were identified as having nutritional risk (NRS-2002 ≥3) upon treatment initiation. There was no correlation between NRS-2002 scores and ECOG PS (Spearman's ρ=0.046; *P*=0.516). In multivariate analysis, NRS-2002 scores (*P*=0.002, HR 2.805, 95%CI: 1.445-5.446) and ECOG PS (*P*=0.015, HR 2.719, 95%CI: 1.218-6.067) were independent prognostic factors for the response to dCRT. NRS-2002 scores (OS: HR 1.530, 95%CI 1.059-2.209; *P*=0.023; PFS: HR 1.517, 95%CI 1.105-2.082; *P*=0.010) and ECOG PS (OS: HR 1.729, 95%CI 1.185-2.522; *P*=0.005; PFS: HR 1.678, 95%CI 1.179-2.387; *P*=0.004) were both independent prognostic factors for OS and PFS. In conclusions, NRS-2002 scores and ECOG PS scales both have prognostic effects on clinical response and survival in LAEC, but a significant association of NRS-2002 scores and ECOG PS were not observed.

## INTRODUCTION

Esophageal carcinoma is an aggressive disease and is often diagnosed at an advanced stage. Dysphagia has been found to be the primary symptom in more than 90% patients,[1] and 40%-80% patients experienced malnutrition at initial diagnosis.[2] Moreover, along with the depletion of nutrition, it increases the individual's risk of having poor performance status (PS).[3] As such, patients with esophageal cancer are potentially at high risk of poor treatment outcomes because of preexisting malnutrition and physical deconditioning.[4]

Treatments for esophageal cancer are commonly multimodal, incorporating surgery, chemotherapy (CT) and radiation therapy (RT). Definitive concurrent chemoradiotherapy (dCRT) is recommended as a preferred curative treatment option for unresectable locally advanced esophageal cancer (LAEC) based on the landmark results of the Radiation Therapy Oncology Group (RTOG) 85-01 trial, in which the long-term follow-up result showed a 5-year overall survival (OS) rate of 26% in the dCRT group compared with 0% in the RT alone group.[5]

In LAEC, there is a paucity of studies clarifying the combined effect of nutritional risk screening 2002 (NRS-2002) scores and Eastern Cooperative Oncology Group (ECOG) PS (also called the WHO or Zubrod score) in predicting treatment response and survival outcomes. A better understanding of these specific indicators is necessary to improve the patient's compliance, increase the therapeutic ratio, reduce the toxic reactions, and compare the results across different studies. Therefore, the purposes of this study were: (1) to evaluate the correlation of baseline NRS-2002 scores and ECOG PS; (2) to determine the impact of NRS-2002 scores and ECOG PS on clinical response; (3) to assess the prognostic effects of NRS-2002 scores and ECOG PS on overall survival (OS) and progression-free survival (PFS) and further provide information for making decisions about dCRT in patients with unresectable LAEC.

## RESULTS

### Patient characteristics

Among 282 unresectable LAEC patients treated with dCRT from 2011 to 2015, 202 patients who fulfilled the criteria were included in the present study. Table 1 summarizes their baseline clinical characteristics. The median age at diagnosis was 58 years (range, 23-76 years), and 147 patients were male while 55 were female. Approximately 85.6% (n=173) of patients were noted to have stages III-IVa. The majority of patients (71.8%) had an ECOG PS score of 0-1. The median value of BMI was 20.60 kg/m^2^ (15.43-27.11 kg/m^2^). A total of 43 patients (21.3%) were classified as underweight (<18.5 kg/m^2^), 123 patients (60.9%) were normal weight (18.5-22.9 kg/m^2^), 36 patients (17.8%) were overweight and obese (≥23.0 kg/m^2^). The median serum albumin level was 37.8 g/L (range, 31.9-49.0 g/L) at baseline.

**Table 1:** Baseline clinical characteristics

Factor	N = 202	Percentage (%)
Age (years)		
Median (range)	58 (23-76)	
Sex		
Female	55	27.2
Male	147	72.8
ECOG PS		
0-1	145	71.8
2	57	28.2
Body mass index (BMI, Kg/m^2^)		
BMI <18.5	43	21.3
18.5≤BMI <22.9	123	60.9
BMI≥23	36	17.8
Albumin (g/L)		
Median (range)	37.8 (31.9-49.0)	
<35	38	18.8
≥35	164	81.2
Pretreatment NRS-2002		
1-2	96	47.5
≥3	106	52.5
T stage		
T_3_	83	41.1
T_4_	119	58.9
N stage		
N_0_	71	35.1
N_1_	131	64.9
M stage		
M_0_	129	63.9
M_1a_	73	36.1
Clinical stage (AJCC 2002)		
T_3_N_0_M_0_	29	14.4
T_3_N_1_M_0_	30	14.8
T_4_N_0_M_0_	23	11.4
T_4_N_1_M_0_	47	23.3
_Any_T_Any_NM_1a_	73	36.1
Histopathology		
SCC	172	85.1
AC	30	14.9
Differentiation		
Well	47	23.3
Fairly	73	36.1
Poorly	82	40.6
Location		
Upper 1/3	59	29.2
Middle 1/3	85	42.1
Lower 1/3	58	28.7
Largest tumour dimension (cm)		
≤4.5	50	24.8
>4.5	152	75.2
CT regimen		
5-Fu+Cisplatin (PF)	110	54.5
Paclitaxel+Cisplatin (TP)	92	45.5
RT delivery		
3D-CRT	132	65.3
IMRT	70	34.7

ECOG PS: Eastern Cooperative Oncology Group performance status; BMI: Body mass index; NRS-2002: Nutritional Risk Screening 2002; AJCC: American Joint Committee on Cancer; SCC: Squamous cell carcinoma; AC: adenocacinoma; 3D-CRT: three-dimensional conformal radiotherapy; IMRT: Intensity-modulated radiation therapy.

52.5% (n=106) of patients were identified as having nutritional risk (NRS-2002 ≥3) at treatment initiation. The maximum nutritional interventions are summarized in Table 2. Only 9.9% (n=20) of patients received diet counseling during the entire dCRT course. 36.1% (n=73) of patients had enteral nutrition (EN) support while 16.8% (n=34) of patients received parenteral nutrition (PN) support. Furthermore, 19.8% (n=40) of patients did not complete the dCRT schedule and 33.7% (n=67) of patients were documented to have grade ≥3 treatment-related toxicities.

**Table 2:** Maximum nutritional intervention during dCRT and therapeutic measures after dCRT failure

Nutritional support	N = 202 (%)
Diet counseling	20 (9.9)
Oral supplements	51 (25.3)
Enteral nutrition (EN)	73 (36.1)
Parenteral nutrition (PN)	34 (16.8)
EN+PN	13 (6.4)
Unknown	11 (5.5)
Chemotherapy	69 (34.2)
Second course RT	21 (10.4)
Brachytherapy	14 (6.9)
Self-expanding metal stent	27 (13.4)
Salvage esophagectomy	16 (7.9)
Best support care	43 (21.3)
Unknown	12 (5.9)

Spearman's rank test was used to determine whether NRS-2002 scores and ECOG PS scales were correlated. A Spearman's ρ of 0.046 (*P*=0.516) suggested that NRS-2002 scores and ECOG PS were not correlated with one another. Additionally, except for ECOG PS, which had a significantly lower correlation with grade ≥3 toxicities (ρ=0.493; *P*<0.001), neither NRS-2002 scores nor ECOG PS scales were highly correlated with other treatment factors (Supplementary Table 1).

### Predictive factors for the response to dCRT

The clinical response was documented according to RECIST. Complete response (CR) was observed in 47 (23.3%) patients, partial response (PR) in 66 (32.6%) patients, stable disease (SD) and progressive disease (PD) in 89 (44.1%) patients, which yielded a clinical response rate (RR) of 55.9% (Table 3). RR was observed in 65 (32.1%) patients who had NRS-2002 scores of 1-2, in 48 (23.8%) patients who had NRS-2002 scores over 3. There was a significant difference in RR between the two groups of patients with different NRS-2002 scores (*P*=0.001). In patients with ECOG PS scales of 0-1, RR was observed in 94 (46.5%) patients, in patients with ECOG PS of 2, RR was observed in 19 (9.4%) patients. There was also a significant difference in RR between the two groups of patients with different ECOG PS scales (*P*<0.001). Univariate analysis of predictive factors of the response to dCRT showed that ECOG PS (HR 3.686, 95%CI 1.929-7.045; *P*<0.001) and NRS-2002 scores (HR 2.534, 95%CI 1.427-4.497; *P*=0.001) were strongly correlated with a better clinical response. Other variables associated with RR were the clinical stage (HR 1.612, 95%CI 1.054-2.463; *P*=0.028), tumor length (HR 2.214, 95%CI 1.118-4.384; *P*=0.023) and dCRT break (HR 3.350, 95%CI 1.609-6.697; *P*=0.001). Multivariate analysis identified that NRS-2002 scores (HR 2.805, 95%CI: 1.445-5.446; *P*=0.002), ECOG PS (HR 2.719, 95%CI: 1.218-6.067; *P*=0.015) and dCRT break (HR 3.323, 95%CI: 1.440-7.672; *P*=0.005) were independent prognostic factors for the clinical response to dCRT (Table 4).

**Table 3:** Clinical response rate of patients with different NRS-2002 scores and ECOG PS scales

	Responder (CR+PR) N (%)	Non-responder (SD+PD) N (%)	P value
Total	113 (55.9)	89 (44.1)	-
*NRS-2002 1-2*	65 (32.1)	31 (15.4)	0.001
*NRS-2002 ≥3*	48 (23.8)	58 (28.7)	
*ECOG PS 0-1*	94 (46.5)	51 (25.2)	< 0.001
*ECOG PS 2*	19 (9.4)	38 (18.9)	

**Table 4:** Predictive factors of clinical response to dCRT in univariate and multivariate analysis

Factor	Univariate		Multivariate	
	P value	HR (95% CI)	P value	HR (95% CI)
Age (<58 vs. ≥58)	0.322	0.755 (0.432-1.317)	-	
Sex (Male/Female)	0.807	1.018 (0.580-2.015)	-	
ECOG PS (0-1 vs. 2)	<0.001	3.686 (1.929-7.045)	0.015	2.719 (1.218-6.067)
BMI (<18.5 vs. 18.5≤BMI <22.9 vs. ≥23)	0.045	0.626 (0.397-0.989)	0.540	0.849 (0.502-1.435)
Albumin (<35 vs. ≥35)	0.321	1.445 (0.698-2.989)	-	
Pretreatment NRS-2002 (1-2 vs. ≥3)	0.001	2.534 (1.427-4.497)	0.002	2.805 (1.445-5.446)
T stage (T3/T4)	0.110	1.595 (0.900-2.825)	-	
N stage (N0/N1)	0.118	1.604 (0.887-2.899)	-	
M stage (M0/M1a)	0.086	1.661 (0.931-2.963)	0.383	0.549 (0.143-2.112)
Clinical Stage (II/III/IVa)	0.028	1.612 (1.054-2.463)	0.126	2.171 (0.805-5.853)
Histopathology (SCC/AC)	0.135	1.816 (0.830-3.974)	-	
Differentiation (Well/Fairly/Poorly)	0.406	1.164 (0.814-1.665)	-	
Location (Upper 1/3 vs. middle 1/3 vs. lower 1/3)	0.312	1.208 (0.837-1.743)	-	
Length (<4.5 vs. ≥4.5)	0.023	2.214 (1.118-4.384)	0.116	1.838 (0.861-3.921)
CT regimen (PF/TP)	0.895	1.038 (0.595-1.814)	-	
RT delivery (3D-CRT/IMRT)	0.521	1.210 (0.676-2.167)	-	
dCRT break (No/yes)	0.001	3.350 (1.609-6.697)	0.005	3.323 (1.440-7.672)
Grade ≥3 toxicity (No/yes)	0.052	1.797 (0.994-3.246)	0.607	1.226 (0.564-2.667)

HR, hazard ratio; CI, confience interval.

### Predictive factors for OS and PFS

**Figure 1:**
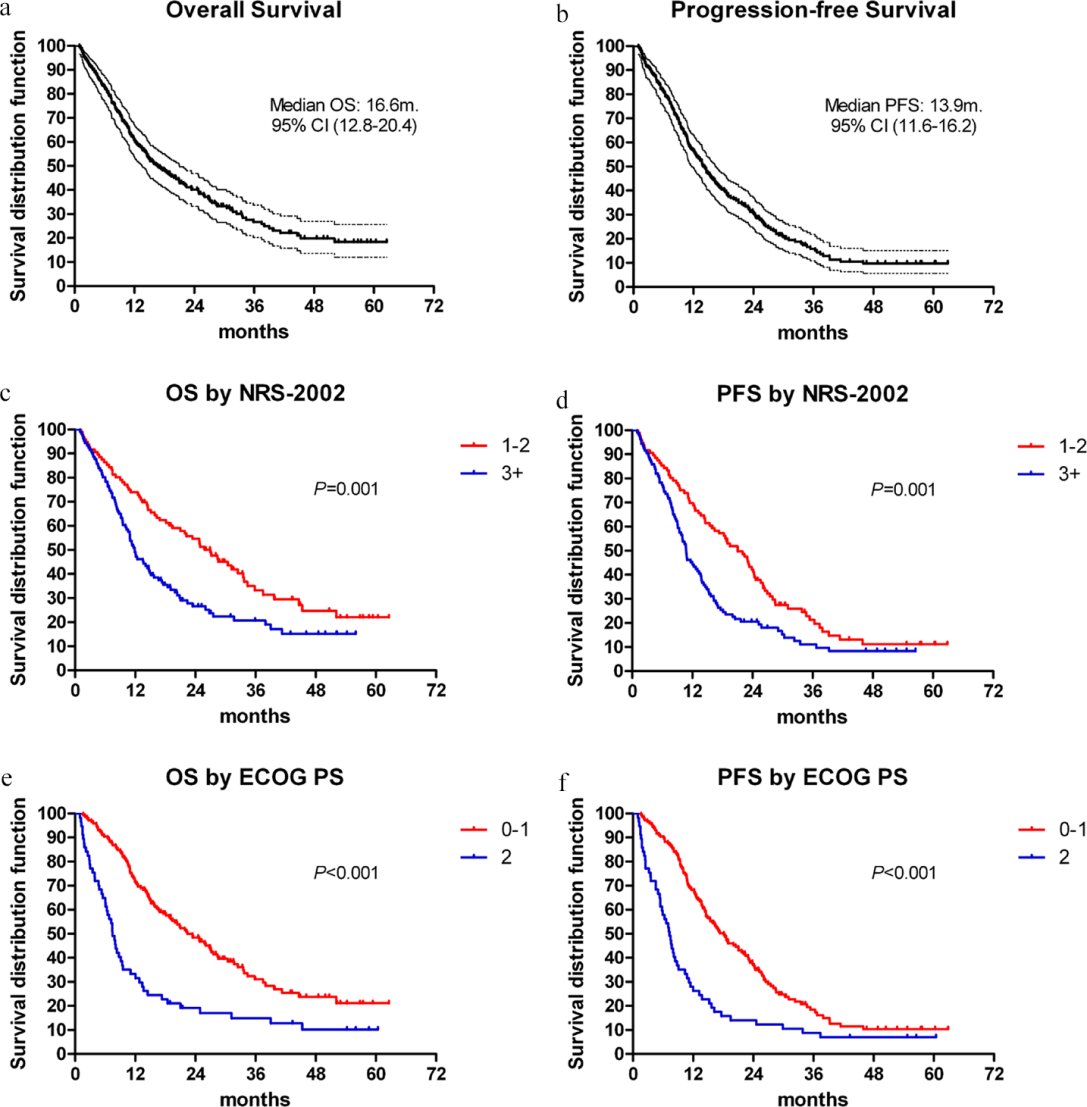
**(a, b)** Overall survival (OS) and progression-free survival (PFS) with 95% confidence interval for LAEC patients treated with dCRT. **(c, d)** OS and PFS curves according to NRS-2002 scores. **(e, f)** OS and PFS curves according to ECOG PS scales.

The median duration of follow-up was 22.7 months (0.9-62.3 months). The median OS was 16.60 ± 1.95 months (95%CI: 12.8-20.4). The 1- and 3-year OS rates were 60.5% (95%CI: 0.538-0.672) and 26.3% (95%CI: 0.194-0.332), respectively. During the follow-up period, 84.7% (n=171) of patients had progressive diseases and the subsequent therapeutic measures were listed in Table 2. The median PFS was 13.90 ± 1.17 months (95%CI: 11.6-16.2). The 1- and 3-year PFS rates were 56.2% (95%CI: 0.493-0.631) and 15.3% (95%CI: 0.098-0.208), respectively. The OS and PFS curves with 95%CI are shown in Figure 1a, 1b. In patients classified to be at nutritional risk at baseline (NRS-2002 ≥3), the median OS and PFS time were 11.8 ± 1.2 months (95%CI: 9.6-14.1) and 10.7 ± 0.9 months (95%CI: 9.1-12.4), respectively. The corresponding median OS and PFS time for patients who had NRS-2002 scores of 1-2 were: 27.0 ± 3.5 months (95%CI: 20.0-33.9) and 21.0 ± 2.2 months (95%CI: 16.7-25.3), respectively. There were significant differences in OS and PFS between the two groups (both *P*=0.001, Figure 1c, 1d). The median OS for patients with ECOG PS scales of 0-1 and 2 prior to receiving dCRT was 22.4 ± 2.8 months (95%CI: 16.9-27.9) and 7.5 ± 0.7 months (95%CI: 6.1-8.8), respectively. The median PFS for patients who were evaluated with ECOG PS scales of 0-1 and 2 was 18.1 ± 2.0 months (95%CI: 14.2-22.0) and 7.3 ± 0.8 months (95%CI: 5.8-8.8), respectively. There were also significant differences between the two groups in OS and PFS according to ECOG PS scales before treatment initiation (both *P*<0.001, Figure 1e, 1f).

As shown in Table 5, univariate analysis revealed that ECOG PS (HR 2.380, 95%CI 1.685-3.363; *P*<0.001), NRS-2002 score (HR 1.739, 95%CI 1.248-2.421; *P*=0.001), T stage (HR 1.762, 95%CI 1.252-2.479; *P*=0.001), N stage (HR 1.520, 95%CI 1.066-2.168; *P*=0.021), M stage (HR 1.880, 95%CI 1.351-2.617; *P*<0.001), clinical stage (HR 1.757, 95%CI 1.368-2.257; *P*<0.001), differentiation (HR 1.254, 95%CI 1.012-1.553; *P*=0.038), tumor length (HR 1.518, 95%CI 1.020-2.258; *P*=0.039) and clinical response (HR 3.215, 95%CI 2.279-4.535; *P*<0.001) were potentially prognostic factors for OS. The variables significantly associated with the PFS were: ECOG PS (HR 2.149, 95%CI 1.548-2.983; *P*<0.001), NRS-2002 score (HR 1.653, 95%CI 1.219-2.241; *P*=0.001), T stage (HR 1.570, 95%CI 1.152-2.141; *P*=0.004), M stage (HR 1.913, 95%CI 1.405-2.605; *P*<0.001), clinical stage (HR 1.671, 95%CI 1.328-2.102; *P*<0.001), differentiation (HR 1.252, 95%CI 1.028-1.526; *P*=0.026) and clinical response (HR 2.703, 95%CI 1.973-3.702; *P*<0.001). The variables associated with OS and PFS in the multivariable Cox model were: NRS-2002 score (OS: HR 1.530, 95%CI 1.059-2.209; *P*=0.023; PFS: HR 1.517, 95%CI 1.105-2.082; *P*=0.010), ECOG PS (OS: HR 1.729, 95% CI 1.185-2.522; *P*=0.005; PFS: HR 1.678, 95% CI 1.179-2.387; *P*=0.004) and clinical response (OS: HR 2.260, 95% CI 1.550-3.296; *P*<0.001; PFS: HR 2.109, 95% CI 1.498-2.968; *P*<0.001).

**Table 5:** Predictive factors of overall survival and progression-free survival in univariate and multivariate analysis

Factor	Overall survival				Progression-free survival			
	Univariate		Multivariate		Univariate		Multivariate	
	P value	HR (95% CI)	P value	HR (95% CI)	P value	HR (95% CI)	P value	HR (95% CI)
Age (<58 vs. ≥58)	0.502	1.118 (0.808-1.546)	-		0.492	1.111 (0.823-1.500)	-	
Sex (Male/Female)	0.664	1.085 (0.751-1.566)	-		0.249	1.216 (0.872-1.696)	-	
ECOG PS (0-1 vs. 2)	<0.001	2.380 (1.685-3.363)	0.005	1.729 (1.185-2.522)	<0.001	2.149 (1.548-2.983)	0.004	1.678 (1.179-2.387)
BMI (<18.5 vs. 18.5≤BMI <22.9 vs. ≥23)	0.053	0.768 (0.589-1.003)	0.789	0.963 (0.729-1.271)	0.161	0.840 (0.658-1.072)	-	
Albumin (<35 vs. ≥35)	0.704	1.086 (0.710-1.659)	-		0.743	1.067 (0.723-1.575)	-	
Pretreatment NRS-2002 (1-2 vs. ≥3)	0.001	1.739 (1.248-2.421)	0.023	1.530 (1.059-2.209)	0.001	1.653 (1.219-2.241)	0.010	1.517 (1.105-2.082)
T stage (T_3_/T_4_)	0.001	1.762 (1.252-2.479)	0.153	1.337 (0.898-1.992)	0.004	1.570 (1.152-2.141)	1.174	1.288 (0.894-1.857)
N stage (N_0_/N_1_)	0.021	1.520 (1.066-2.168)	0.658	1.100 (0.722-1.676)	0.070	1.345 (0.977-1.854)	0.897	1.026 (0.695-1.515)
M stage (M_0_/M_1a_)	<0.001	1.880 (1.351-2.617)	0.897	0.940 (0.369-2.395)	<0.001	1.913 (1.405-2.605)	0.521	1.310 (0.574-2.986)
Clinical Stage (II/III/IV_a_)	<0.001	1.757 (1.368-2.257)	0.234	1.604 (0.737-3.488)	<0.001	1.671 (1.328-2.102)	0.517	1.245 (0.642-2.413)
Histopathology (SCC/AC)	0.304	1.254 (0.814-1.930)	-		0.689	1.089 (0.717-1.655)	-	
Differentiation (Well/Fairly/Poorly)	0.038	1.254 (1.012-1.553)	0.434	1.096 (0.871-1.380)	0.026	1.252 (1.028-1.526)	0.300	1.117 (0.906-1.378)
Location (Upper 1/3 vs. middle 1/3 vs. lower 1/3)	0.409	0.912 (0.732-1.136)	-		0.199	0.875 (0.713-1.073)	-	
Length (<4.5 vs. ≥4.5)	0.039	1.518 (1.020-2.258)	0.292	1.245 (0.829-1.870)	0.163	1.282 (0.904-1.817)	-	
CT regimen (PF/TP)	0.612	1.088 (0.785-1.507)	-		0.885	1.023 (0.755-1.384)	-	
RT delivery (3D-CRT/IMRT)	0.804	0.958 (0.681-1.346)	-		0.724	0.945 (0.689-1.295)	-	
dCRT break (No/yes)	0.063	1.451 (0.980-2.148)	0.322	1.234 (0.814-1.872)	0.135	1.327 (0.915-1.925)	-	
Grade ≥3 toxicity (No/yes)	0.405	1.155 (0.823-1.619)	-		0.529	1.106 (0.807-1.516)	-	
Clinical response (CR+PR vs SD+PD)	<0.001	3.215 (2.279-4.535)	<0.001	2.260 (1.550-3.296)	<0.001	2.703 (1.973-3.702)	<0.001	2.109 (1.498-2.968)

HR, hazard ratio; CI, confience interval.

## DISCUSSION

In this study, our results suggest that increased nutritional risk scores based on NRS-2002 and impaired ECOG PS at baseline are associated with a significant poor clinical response and decreased survivals (both OS and PFS) in unresectable LAEC patients who received dCRT. Furthermore, our study shows that NRS-2002 scores and ECOG PS scales were not correlated with one another.

A series of studies in the literature have underlined that nutritional parameters can have independent prognostic effects on treatment outcomes of esophageal cancer. In a recently published post hoc analysis of the SCOPE1 trial,[6] 258 patients were randomly assigned to receive dCRT based on cisplatin and capecitabine ± cetuximab. Nutritional Risk Index (NRI) scores were collected based on the following formula: *NRI*=(1.519×albumin g/dl)+41.7(present weight/ideal weight). An NRI score <100 was identified as patients at nutritional risk. Nutritional interventions included dietary advice, oral supplementation and/or major intervention (enteral feeding/tube placement). With a median follow-up time of 25.0 months, their results showed that baseline NRI <100 was strongly predicted for reduced OS (HR 12.45, 95%CI 5.24-29.57; *P*<0.001) and positive nutritional intervention at baseline improved OS (dietary advice (HR 0.12, *P*=0.004), oral supplementation (HR 0.13, *P*<0.001) or major intervention (HR 0.13, *P*=0.003)). In our study, increased NRS-2002 scores (≥3) were also associated with impaired survival outcomes (OS (HR 1.530, *P*=0.023); PFS (HR 1.517, *P*=0.010)), which was consistent with their findings. In addition, this study further showed that patients with increased NRS-2002 scores had significantly decreased clinical response (32.1% *vs.* 23.8%; *P*=0.001). Other tools for nutritional screening in cancer patients at present included the subjective global assessment (SGA),[7] the malnutrition universal screening tool (MUST),[8] and the mini nutritional assessment (MNA).[9] With different nutritional screening tools and parameters utilized in the clinic, it raises the question of which should be used as the preferred screening tool to detect malnutrition in cancer patients. NRS-2002 is a recommended instrument by the European Society for Clinical Nutrition and Metabolism (ESPEN) for hospitalized patients [10] and has also been validated in China.[11] Compared to other tools, a previous study has demonstrated that NRS-2002 appeared to be an ideal screening instrument relative to MUST and MNA with a sensitivity and specificity of 77.8% and 80.8%, respectively, for internal medicine, and it was less time consuming and required less examiner training than other tools.[12]

Unlike a number of nutritional screening and assessment tools applied clinically, limited measures of PS are widely used, among them the ECOG PS and the Karnofsky's Scale of Performance Status (KPS). Compared with KPS, a previous study showed that KPS showed a lower ability than ECOG PS to discriminate patients with different prognoses in lung cancer. Considering the necessity of comparing results from different studies based on the “unbiased” foundation, the authors suggested that ECOG PS should be preferred to KPS.[13] In 2014, Clavier *et al.* conducted a retrospective study of 143 esophageal cancer patients undergoing dCRT. With a median follow-up time of 20.8 months, the median OS and disease-free survival (DFS) were 22.1 and 14.9 months, respectively. Multivariate analysis revealed that NRI ≥97.5 (HR 0.68, 95%CI 0.523-0.880; *P*=0.003) and ECOG PS of 0 (HR 0.77, 95%CI 0.628-0.940; *P*=0.011) were independent favorable prognostic factors for OS.[14] Sun *et al.* reported their data from a large-scale retrospective study of 502 esophageal squamous cell carcinoma (ESCC) patients and showed that ECOG PS (unfavorable: ECOG 2; HR 2.809, 95%CI 1.962-4.020; *P*<0.001) was also an independent prognostic factor in multivariate analysis of OS.[15] Our study further identified the ECOG PS less than 1 as the other independent indicator for favorable clinical response in LAEC patients (HR 2.719, 95%CI: 1.218-6.067; *P*=0.015) and better ECOG PS scales evaluated at baseline were also correlated with better clinical response (46.5% vs. 9.4%; *P*<0.001). These results could be explained in part by the poor ECOG PS that may capture disease-related features, such as aggressive tumor biology and inadequate organ reserve, which along with the burden of preexisting malunutrition, might decrease the anti-cancer effects and increase the resistance to dCRT.

The limitations of the present study should be mentioned. First is the nature of its retrospective design and it is associated with potential and unmeasured factors that might exert an influence on the final results. In addition, the assessment of PS is subjective; several studies had indicated that the presence of a systemic inflammatory response criterion, as evidenced by the Glasgow Prognostic Score, appeared to be superior to ECOG PS in predicting the response and survival in thoracic cancer patients.[16, 17]

In conclusion, the current study suggests that both the NRS-2002 scores and ECOG PS provide important, distinct information in predicting clinical response and survival in unresectable LAEC patients treated with dCRT. As such, nutritional assessment and ECOG PS data should both be included in LAEC outcome studies. We expect prospective nutritional intervention studies to improve the therapeutic ratio and survival outcomes in the near future.

## MATERIALS AND METHODS

### Study population

This multicenter retrospective study was conducted between January 2011 and December 2015 at four endemic areas of esophageal cancer in China (Department of Radiation Oncology, Zhejiang Provincial People's Hospital; Department of Radiation Oncology, Ningbo Mingzhou Hospital; Department of Radiation Oncology, Jinhua Guangfu Hospital; Department of Radiation Oncology, Jiangxi Cancer Hospital). This analysis was approved by the Institutional Review Boards (IRBs), with all participating cancer centers providing the necessary institutional data use agreements (Zhejiang Provincial People's Hospital, Ningbo Mingzhou Hospital, Jinhua Guangfu Hospital and Jiangxi Cancer Hospital) and was performed in accordance with the ethical standards of the World Medical Association Declaration of Helsinki. Patients’ records were anonymized and de-identified prior to analysis.

### Eligibility

The inclusion criteria in the present study were as follows: I) cytopathologically confirmed as esophageal malignancy; II) unable or refusing to undergo surgical resection; III) ECOG PS of ≤2; and IV) no uncontrolled serious diseases and adequate organ function. The exclusion criteria were as follows: early-stage esophageal cancer or evidence of distant metastasis at diagnosis, prior administration of surgery or non-cisplatin based chemotherapy, and incomplete data on the treatment response and survivals.

### Pre-treatment work-up

Pre-treatment procedures included complete physical examination, electrocardiography, and blood and pulmonary function tests. Baseline nutritional assessment was operationalized with the NRS-2002 under the recommendation of the ESPEN for hospitalized patients. The NRS-2002 is based on three variables: weight loss, BMI, amount of food intake in the preceding week in addition to the patient's age and the severity of the underlying disease. Patients are classified as being at nutritional risk (score ≥3) or not (score <3) according to the total score obtained.[10, 18] Baseline PS was assessed using the ECOG scale which categorized patients’ level of functioning into 5 levels.[19] A patient's BMI was calculated and classified according to the Asian-specific BMI cutoff values as follows: underweight (<18.5 kg/m^2^); normal weight (18.5-22.9 kg/m^2^); overweight and obese (≥23.0 kg/m^2^).[20] The extent of disease evaluation included endoscopy of the esophagus, barium swallowing, endoscopic ultrasonography, enhanced computed tomography (CT), positron emission tomography/CT (PET/CT, if available), bronchoscopy (to exclude tracheoesophageal fitula) and bone scan (if clinically indicated). Metastatic lymph nodes were defined as ≥1 cm in their greatest diameter on CT imaging. Clinical stages (II-IVa) were diagnosed according to the 2002 American Joint Committee on Cancer staging system (version 6.0, AJCC).

### Treatment schedule

A total of 132 patients (65.3%) received three-dimensional conformal radiotherapy (3D-CRT) and the other 70 patients were treated with intensity-modulated radiation therapy (IMRT). The preplanned radiation dose was 54.0-60.0 Gy, which was given as 30 fractions of 1.8-2.0 Gy each once a day 5 days per week. The definitions of gross tumor volume (GTV), clinical target volume (CTV), planning target volume (PTV) and dose-volume constraints of normal tissues have been described previously.[21] All patients received cisplatin-based chemotherapy combined with 5-fluorouracil (5-Fu) or paclitaxel (PTX). In the PF (5-Fu+Cisplatin) group, 110 patients received two cycles of PF regimen at 4-week intervals. Cisplatin at 75 mg/m^2^ was administered intravenously on Day 1 and Day 29 with standard hydration, followed by 5-Fu at 1000 mg/m^2^ per day administered by continuous intravenous infusion on days 1-4 of each cycle. In the TP (PTX+Cisplatin) group, 92 patients also received the same preplanned dose of cisplatin, followed by PTX at 135 mg/m^2^ *i.v.* administered for 3 hours on day 1 and day 29 with standard premedications. Dose modification of dCRT or suspension of treatment was considered if any grade 4 toxicities occurred and restarted when toxicities recovered to grades ≤2.

### Nutritional support

According to the NRS-2002 suggestion, patients identified as scores of 1-2 received individualized diet counseling and support to help maintain nutritional status, whereas those with risk scores ≥3 received nutritional intervention including oral nutritional supplements, EN, and/or PN. All patients were reviewed weekly throughout the treatment course. Patients who developed severe dysphagia during the treatment course received nasogastric tube placement, depending on the treatment week in which this occurred.

### Treatment assessment and follow-up

Clinical response was assessed according to the RECIST (Response Evaluation Criteria in Solid Tumors) system 6-8 weeks after the completion of treatment. CR was defined as the disappearance of all target lesions on CT images, PR was defined as a ≥30% decrease in the sum of the longest diameter of target lesions, PD was defined as a ≥20% increase in the sum of the longest diameter of target lesions and SD was defined as neither sufficient shrinkage to qualify for PR nor a sufficient increase to qualify for PD.[22] The National Cancer Institute Common Toxicity Criteria (version 3.0) was used to score acute treatment toxicity.[23] Follow-up modalities included physical examination, blood test, upper endoscopy, enhanced CT of the neck (mandatory for cervical EC), chest, abdomen, and pelvis. Follow-up evaluations were performed every 3 months for the first year, every 6 months for the second year, and then on a yearly basis.

### Statistical analysis

The cutoff date of the last follow-up was December 31, 2016 for the censored data analysis. OS was determined as the time that elapsed from the date of dCRT initiation to the last follow-up or to the date of death. Progression-free survival (PFS) was defined as the interval between the first day of treatment and the date of documented failure or the date of the last follow-up for those remaining. Survival curves were generated using the Kaplan-Meier method and compared with the log-rank test. Correlation between the baseline NRS-2002 scores and ECOG PS was estimated using the Spearman rank correlation coefficient (ρ). Response to dCRT was categorized as 1 (CR+PR) and 2 (SD+PD) for the purpose of analysis. A univariate analysis was performed to identify the predictive factors for the response to dCRT on one hand and to OS and PFS on the other hand. Variables identified with a 2-sided *P* value <0.10 on univariate analysis were included in the multivariate analyses. Multivariate analysis of the predictive factors for the response to dCRT was performed using binary logistic regression with calculation of the hazard ratio (HR) and a confidence interval (CI) of 95%. Multivariate analysis of the predictive factors of OS and PFS were performed using a Cox regression model. *P*<0.05 was accepted as statistically significant. All statistical analyses were conducted using IBM SPSS for Windows version 22.0 (SPSS, Armonk, New York, USA).

## SUPPLEMENTARY MATERIALS TABLE


