# Supplementary material for: Pretreatment nutritional risk scores and performance status are prognostic factors in esophageal cancer patients treated with definitive chemoradiotherapy
Source: Oncotarget. 2017 Oct 19;8(58):98974–84. doi: 10.18632/oncotarget.21940 (PMC5716782; doi:10.18632/oncotarget.21940)
Supplement: Supplementary file 1 [file oncotarget-08-98974-s001.pdf]

Pretreatment nutritional risk scores and performance status are prognostic factors in esophageal cancer patients treated with definitive chemoradiotherapy

SUPPLEMENTARY MATERIALS

Supplementary Table 1: Correlation coefficient of NRS-2002 scores and ECOG PS scales

	NRS-2002	ECOG PS
Age (<58 years versus ≥58 years)	0.020	-0.165*
Sex (Male/Female)	-0.131	0.111
ECOG PS (0-1 versus 2)	0.046	1.000
BMI (<18.5 Kg/m2 versus 18.5 Kg/m2≤BMI <22.9 Kg/m2 versus ≥23 Kg/m2)	-0.289**	-0.125
Albumin (<35 g/L versus ≥35 g/L)	-0.002	0.020
NRS-2002 at diagnosis (1-2 versus ≥3)	1.000	0.046
T stage (T3/T4)	0.092	0.188
N stage (N0/N1)	0.047	0.116
M stage (M0/M1a)	0.035	0.147*
Clinical stage	0.042	0.157*
Histopathology (SCC/AC)	0.202**	0.017
Differentiation (Well/Fairly/Poorly)	0.013	0.103
Tumor location (Upper 1/3 versus middle 1/3 versus lower 1/3)	0.059	0.047
Tumor length (<4.5 cm versus ≥4.5cm)	0.028	0.079
CT regimen (5-Fu+cisplatin/Paclitaxel+cisplatin)	0.054	0.023
RT delivery (3D-CRT/IMRT)	0.026	-0.017
dCRT break (No/yes)	-0.050	0.185
Grade ≥3 toxicity (No/yes)	-0.109	0.493**
Tumor response	0.226**	0.286**

Correlation coefficient: *: P<0.05, **: P<0.01.
